# Supplementary material for: OpenMM 7: Rapid development of high performance algorithms for molecular dynamics
Source: PLoS Comput Biol. 2017 Jul 26;13(7):e1005659. doi: 10.1371/journal.pcbi.1005659 (PMC5549999; doi:10.1371/journal.pcbi.1005659)
Supplement: S1 Source Code — (ZIP) [file pcbi.1005659.s002.zip › OpenMM-7.0.1-Source/docs/api-python/generated/simtk.openmm.openmm.RBTorsionForce.html]

RBTorsionForce — OpenMM 7.0.1.dev-5e86c4f documentation


# RBTorsionForce¶

*class* `simtk.openmm.openmm.``RBTorsionForce`(*\*args*)¶
:   This class implements an interaction between groups of four particles that varies with the torsion angle between them according to the Ryckaert-Bellemans potential. To use it, create an RBTorsionForce object then call addTorsion() once for each torsion. After a torsion has been added, you can modify its force field parameters by calling setTorsionParameters(). This will have no effect on Contexts that already exist unless you call updateParametersInContext().

    `__init__`(*self*) → RBTorsionForce¶
    :   \_\_init\_\_(self, other) -> RBTorsionForce

        Create a RBTorsionForce.

    Methods

    |  |  |
    | --- | --- |
    | `__init__`((self) -> RBTorsionForce) | \_\_init\_\_(self, other) -> RBTorsionForce |
    | `addTorsion`((self, particle1, particle2, ...) | Add a Ryckaert-Bellemans torsion term to the force field. |
    | `getForceGroup`((self) -> int) | Get the force group this Force belongs to. |
    | `getNumTorsions`((self) -> int) | Get the number of Ryckaert-Bellemans torsion terms in the potential function |
    | `getTorsionParameters`(self, index) | Get the force field parameters for a Ryckaert-Bellemans torsion term. |
    | `setForceGroup`(self, group) | Set the force group this Force belongs to. |
    | `setTorsionParameters`(self, index, particle1, ...) | Set the force field parameters for a Ryckaert-Bellemans torsion term. |
    | `updateParametersInContext`(self, context) | Update the per-torsion parameters in a Context to match those stored in this Force object. |
    | `usesPeriodicBoundaryConditions`((self) -> bool) | Returns whether or not this force makes use of periodic boundary conditions. |

    `getNumTorsions`(*self*) → int¶
    :   Get the number of Ryckaert-Bellemans torsion terms in the potential function

    `addTorsion`(*self*, *particle1*, *particle2*, *particle3*, *particle4*, *c0*, *c1*, *c2*, *c3*, *c4*, *c5*) → int¶
    :   Add a Ryckaert-Bellemans torsion term to the force field.

        |  |  |
        | --- | --- |
        | Parameters: | - **particle1** (*int*) – the index of the first particle forming the torsion - **particle2** (*int*) – the index of the second particle forming the torsion - **particle3** (*int*) – the index of the third particle forming the torsion - **particle4** (*int*) – the index of the fourth particle forming the torsion - **c0** (*double*) – the coefficient of the constant term, measured in kJ/mol - **c1** (*double*) – the coefficient of the 1st order term, measured in kJ/mol - **c2** (*double*) – the coefficient of the 2nd order term, measured in kJ/mol - **c3** (*double*) – the coefficient of the 3rd order term, measured in kJ/mol - **c4** (*double*) – the coefficient of the 4th order term, measured in kJ/mol - **c5** (*double*) – the coefficient of the 5th order term, measured in kJ/mol |
        | Returns: | the index of the torsion that was added |
        | Return type: | int |

    `getTorsionParameters`(*self*, *index*)¶
    :   Get the force field parameters for a Ryckaert-Bellemans torsion term.

        |  |  |
        | --- | --- |
        | Parameters: | **index** (*int*) – the index of the torsion for which to get parameters |
        | Returns: | - **particle1** (*int*) –   the index of the first particle forming the torsion - **particle2** (*int*) –   the index of the second particle forming the torsion - **particle3** (*int*) –   the index of the third particle forming the torsion - **particle4** (*int*) –   the index of the fourth particle forming the torsion - **c0** (*double*) –   the coefficient of the constant term, measured in kJ/mol - **c1** (*double*) –   the coefficient of the 1st order term, measured in kJ/mol - **c2** (*double*) –   the coefficient of the 2nd order term, measured in kJ/mol - **c3** (*double*) –   the coefficient of the 3rd order term, measured in kJ/mol - **c4** (*double*) –   the coefficient of the 4th order term, measured in kJ/mol - **c5** (*double*) –   the coefficient of the 5th order term, measured in kJ/mol |

    `setTorsionParameters`(*self*, *index*, *particle1*, *particle2*, *particle3*, *particle4*, *c0*, *c1*, *c2*, *c3*, *c4*, *c5*)¶
    :   Set the force field parameters for a Ryckaert-Bellemans torsion term.

        |  |  |
        | --- | --- |
        | Parameters: | - **index** (*int*) – the index of the torsion for which to set parameters - **particle1** (*int*) – the index of the first particle forming the torsion - **particle2** (*int*) – the index of the second particle forming the torsion - **particle3** (*int*) – the index of the third particle forming the torsion - **particle4** (*int*) – the index of the fourth particle forming the torsion - **c0** (*double*) – the coefficient of the constant term, measured in kJ/mol - **c1** (*double*) – the coefficient of the 1st order term, measured in kJ/mol - **c2** (*double*) – the coefficient of the 2nd order term, measured in kJ/mol - **c3** (*double*) – the coefficient of the 3rd order term, measured in kJ/mol - **c4** (*double*) – the coefficient of the 4th order term, measured in kJ/mol - **c5** (*double*) – the coefficient of the 5th order term, measured in kJ/mol |

    `updateParametersInContext`(*self*, *context*)¶
    :   Update the per-torsion parameters in a Context to match those stored in this Force object. This method provides an efficient method to update certain parameters in an existing Context without needing to reinitialize it. Simply call setTorsionParameters() to modify this object’s parameters, then call updateParametersInContext() to copy them over to the Context.

        The only information this method updates is the values of per-torsion parameters. The set of particles involved in a torsion cannot be changed, nor can new torsions be added.

    `usesPeriodicBoundaryConditions`(*self*) → bool¶
    :   Returns whether or not this force makes use of periodic boundary conditions.

        |  |  |
        | --- | --- |
        | Returns: | true if force uses PBC and false otherwise |
        | Return type: | bool |

    `__delattr__`¶
    :   x.\_\_delattr\_\_(‘name’) <==> del x.name

    `__format__`()¶
    :   default object formatter

    `__getattribute__`¶
    :   x.\_\_getattribute\_\_(‘name’) <==> x.name

    `__hash__`¶

    `__reduce__`()¶
    :   helper for pickle

    `__reduce_ex__`()¶
    :   helper for pickle

    `__sizeof__`() → int¶
    :   size of object in memory, in bytes

    `__str__`¶

    `getForceGroup`(*self*) → int¶
    :   Get the force group this Force belongs to.

    `setForceGroup`(*self*, *group*)¶
    :   Set the force group this Force belongs to.

        |  |  |
        | --- | --- |
        | Parameters: | **group** (*int*) – the group index. Legal values are between 0 and 31 (inclusive). |

High performance molecular simulation on GPUs


### Navigation

- Application Layer
- Library Layer
  - Core Objects
  - Forces
  - Integrators
  - Extras

©2015, Stanford University and the Authors.
|
Powered by Sphinx 1.3.1
& Alabaster 0.7.7
|
Page source
